# Are integrated care models associated with improved drug safety in Swiss primary care? an observational analysis using healthcare claims data

**DOI:** 10.1371/journal.pone.0311099

**Published:** 2024-09-26

**Authors:** Renato Farcher, Sereina M. Graber, Stefan Boes, Carola A. Huber

**Affiliations:** 1 Department of Health Sciences, Helsana Group, Zürich, Switzerland; 2 Department of Health Sciences and Medicine and Center for Health, Policy and Economics, University of Lucerne, Lucerne, Switzerland; 3 Institute of Primary Care, University of Zürich, University Hospital Zürich, Zürich, Switzerland; Ekiti State University College of Medicine, NIGERIA

## Abstract

**Background:**

Integrated care models (ICMs) might be an effective strategy to improve patients’ quality of care. The aim of this study was to compare different ICMs such as family-doctor models, and a standard care model (SCM) regarding patients’ drug safety in Swiss primary care.

**Methods:**

We performed an observational study using health insurance claims data from patients who were continuously enrolled in an ICM or in a SCM between 2020 and 2021. ICMs included family-doctor model (FDM), family-doctor model light (FDM-light) and the telemedicine model (TM). Drug safety was assessed by the prescription of potentially inappropriate proton pump-inhibitors (PIPPI), opioids (PIO), medications (PIM), and polypharmacy. Propensity-score-weighted multiple logistic regression models were used to examine the association between different types of ICMs and drug safety.

**Results:**

Patients in FDM had significantly lower odds of receiving PIPPI (OR, 0.86; CI 95%, 0.83–0.89), PIO (OR, 0.81; CI 95%, 0.76–0.85), PIM (OR, 0.94; CI 95%, 0.91–0.97), and polypharmacy (OR, 0.94; CI 95%, 0.91–0.97) compared to patients in SCM. Potentially inappropriate prescribing was also lower in patients in TM and partly in FDM-light than in SCM. Persons enrolled in FDM were less likely to receive PIM (OR, 0.93; CI 95%, 0.89–0.97) and polypharmacy (OR, 0.94; CI 95%, 0.90–0.99) than those in FDM-light, whereas the odds of receiving PIPPI and polypharmacy were higher in FDM than in TM.

**Conclusion:**

ICMs were significantly associated with higher drug safety compared to SCM for most outcomes. Findings suggest that patients may benefit most from ICMs with a high degree of coordination or gatekeeping. ICM may represent an effective approach to improve patients’ drug safety and, thus, to reduce the risk of adverse events.

## Background

Medication is the main therapy of modern healthcare for patients in developed countries. In Switzerland, over 120 million medications are prescribed to around 6.5 million people every year. In 2021, the medication costs amounted to CHF 8.1 billion, which corresponded to more than 22% of the total healthcare costs [1,2]. Appropriately used medications can prevent or delay the onset or the progression of diseases and can significantly improve the quality of life of those affected. Used inappropriately, they can increase treatment burden and lead to adverse health outcomes such as comorbidities, hospitalizations, or death which again are associated with higher costs and lower quality of life [3]. Thus, patients’ drug safety is an important therapeutic target in high-quality healthcare systems and reflects appropriate medication prescribing in daily clinical practice. However, evidence suggests a high proportion of potentially inappropriate prescribing in general [4,5] and especially in the elderly population [6]. For example, proton pump inhibitors (PPIs) are one of the most prescribed medications worldwide and their inappropriate use is reported commonly in the general population. A German study revealed that 35% of patients received a PPI prescription without reasonable indication [7] and a Swiss study showed that 25% received a cumulative PPI dose not supported by medical guidelines [8]. Similar results were observed in other international studies [9,10]. Another example of inappropriate prescribing is the prescription of strong opioids such as fentanyl or oxycodone in non-cancer patients. Inappropriate prescribing was reported in the US [11] and other countries [12,13] with studies also demonstrating association to opioid-related mortality [11,12]. While the public health burden of inappropriate opioids prescription is still relative low in Switzerland, evidence revealed a concerning increase of potentially inappropriate opioids (PIO) prescriptions as well [14,15]. Moreover, inappropriate prescribing is especially a major risk for elderly patients. A recent systematic review of 94 studies revealed that almost 40% of the elderly received medications that were explicitly not recommended for this patient group due to potentially severe adverse events [6]. Further, studies in the elderly population demonstrated a high prevalence of polypharmacy [16], defined as the simultaneous use of multiple medications, and also showed an increased risk of hospitalization [17] and mortality [18] with rising number of prescribed medications.

Integrated care models (ICMs) might be effective strategies to improve patients’ drug safety. ICMs are approaches to integrated care focusing on the improvement of quality of care and reduction of unnecessary healthcare costs. They often include features such as limited network of contracted healthcare providers and elements on quality management and financing [19]. While ICMs were implemented in early 1980 in the US, European countries such as Netherlands, Germany or Switzerland implemented ICMs much later [20]. The models differ in structure, focus, and implementation and are adapted to their specific healthcare systems needs and challenges. For example, in Germany, the insurers are obliged to have selective contracts with physician networks and provide general practitioner (GP)-centered healthcare, where the GP acts as the first provider and coordinator of care [21]. In Switzerland, insurers are permitted to offer different types of ICMs for their mandatory health insurance coverage, ranging from a family-doctor model (FDM) to a remote telemedicine model (TM) with different degrees of continuity of care and gatekeeping. Despite the increasing number of persons choosing ICMs as their health insurance coverage [22], evidence of different ICMs and their association with drug safety in Switzerland remains scarce and outdated [23,24]. Few studies from Switzerland showed that patients enrolled in ICMs were not only less likely to receive potentially inappropriate medication (PIM) and antibiotics [23] but also different other active agents [24]. There is also only little international evidence, which mainly investigated the effect of integrated care interventions on drug safety [25,26] or evaluated different healthcare delivery models (e.g. telemedicine) without considering the concept of integrated care [27,28].

Therefore, the aim of this study was to compare different types of ICMs and SCM regarding various drug safety outcomes in the Swiss primary care setting. Drug safety was measured by the prescription of PIPPIs, PIOs and PIMs as well as polypharmacy using a large Swiss claims database.

## Methods

### Study design and study populations

We performed a retrospective observational cohort study based on healthcare claims data from one of the largest health insurance companies in Switzerland, the Helsana Group. The anonymized data covers more than one fifth of the total population with mandatory health insurance (around 1.4 million persons) from all geographical regions of Switzerland. Claim files were recorded at the patient-level and contained information on prescribed drugs (including substance and dose), received in- and outpatient medical services and laboratory tests as well as information on the chosen health insurance plan and deductible level. In addition, the database contained sociodemographic information on the enrollees such as age, sex, and region of living. The study population includes patients with compulsory health insurance who were continuously enrolled in one of the given health insurance models between 2020 and 2021. We excluded patients who were deceased, pregnant, lived abroad or in a nursing home and who were younger than 18 or older than 85 years. For the analysis, three samples for the corresponding drug safety outcomes were generated by applying outcome-specific in- and exclusion criteria.

### Health insurance models

In Switzerland, basic health insurance is mandatory, and every resident is free to choose among the SCM and different ICM models. Insured persons cannot be enrolled in more than one insurance model at a time, as they are mutually exclusive.

The SCM is characterized by direct and unlimited access to any physician in the outpatient setting. In contrast, ICMs are defined by the following characteristics of IC: first, gatekeeping at the beginning of and second, coordination during the treatment process. According to the Swiss Federal Law on Health Insurance (KVG/LAMal), there is no reimbursement of IC services in the Swiss healthcare system. However, physician networks, which comprise independent physicians and physicians of health maintenance organizations (HMOs), are allowed to negotiate a cooperation agreement with health insurances for patients who are enrolled in an ICM. The agreement defines the reimbursement for IC services and regulates the type of cooperation. The different types of ICMs were categorized as follows:

**FDM:** Patients in FDM choose a primary care physician from a list of selectable physicians as their first provider and coordinator of care. The treating physician is part of a physician network, which has a cooperation agreement with the health insurance. These physicians commit to a network-specific quality standard and provide, for example, periodic quality circles or medical education for their employees.

**FDM-light:** Patients in FDM-light also choose a primary care physician from a list of selectable physicians as their first provider and coordinator of care. In contrast to patients enrolled in FDM, the selected physician is not necessarily part of an approved physician-network. There is no cooperation agreement with the health insurance company, due to insufficient quality standards of the physician-network.

**TM:** When medical care is needed, patients in TM call an independent medical center, which offers medical support at any time. The first provider of care is mostly a healthcare professional (e.g., nurse), who coordinates the further treatment process to which the patient is obliged to follow. If medically necessary, the patient is referred to a TM physician or to another physician for an in-person consultation.

### Drug safety

We selected four drug safety quality outcomes: PIPPI, PIO, PIM, and polypharmacy. The selection is based on previous findings showing 1) a high relevance in the Swiss healthcare system, 2) reliable operationalization with claims data, and 3) high rates of potentially inappropriate prescribing in both the general and the older population. The outcomes are previously defined primary care related Swiss quality indicators [29,30] and were determined at the patient level for 2021. The identification of prescribed medications was based on the WHO Anatomical Therapeutic Chemical (ATC) classification system [31]. PIPPI assess patients who were potentially inappropriately treated with PPI and were measured in a sample of patients with ≥1 PPI prescription. Inappropriate dosage followed the definition of Muheim et al. [8], defined by a minimum of 11.5 g pantoprazole dose equivalents during 365 consecutive days. PIOs were evaluated based on a patient sample with ≥2 prescriptions of pain medications with a prescription gap of ≥4 weeks (also across quarters) per quarter year. Cancer patients, patients in palliative care or opioid substitution programs were excluded. PIOs were defined by the prescription of ≥2 weak or strong opioids with a prescription gap of ≥4 weeks per quarter year. PIM and polypharmacy (prescription of ≥5 different active ingredients, i.e. ATC codes) represent drug safety measures in patients aged 65 or older with ≥1 medication prescription in the given year. They were evaluated per quarter year and aggregated at the annual level (if ≥1 PIM or polypharmacy per year). PIMs were assessed based on the Beers [32] and PRISCUS [33] criteria and include medications, which should be avoided in elderly persons due to increased risks of adverse events and lack of evidence of effectiveness where alternative medication is available [34]. Detailed information on the four drug safety outcomes is given in S1 Table.

### Covariates

The analyses included the type of health insurance model as the main predictor of interest, and patients’ sociodemographic (age, sex, and language region) and morbidity characteristics as covariates. Morbidity measures were assessed in 2020, one year prior to the observation year of the outcome measure. It included the number of chronic diseases, hospitalization (≥ 1 overnight stay), and number of outpatient consultations (primary care and specialist physician). The type of health insurance model and all other covariates were obtained from the observation year 2021. The number of chronic diseases was assessed using pharmaceutical cost groups (PCGs). PCGs are based on prescribed medication data and are a well-established and reliable pharmacy-based mapping approach for the assessment of multimorbidity when diagnoses are missing [35]. The number of PCGs were grouped in four categories: 0–1, 2–3, 4–5, ≥ 6.

### Statistical analysis

Patient characteristics of the corresponding sample (one each for the four outcomes of interest) and prevalence of drug safety outcomes by type of health insurance model were presented using descriptive statistics. Frequencies and percentages were used for categorical and means and standard deviations for continuous variables. We used binary logistic regression models and calculated corresponding odds ratios (OR) to test for the association between the health insurance models and the drug safety outcomes. To balance the differences in baseline characteristics between patients enrolled in the different health insurance models, the regression models were weighted based on propensity scores [36], and additionally adjusted for covariates referred to as the doubly robust approach. To calculate the propensity scores, we estimated multinomial regression models with sociodemographic characteristics and proxies of morbidity as independent variables and the indicator variable for health insurance models as the dependent variable. The package WeightIt [37] was used for the propensity score model and resulting weights, and the survey package [38] to estimate the weighted logistic regressions and robust standard errors. In addition, the models were rerun without propensity score weighting (1) using as a simple logistic regression with the type of health insurance model as the single predictor variable, and (2) using traditional covariate adjustment via multiple regression analyses (results shown in S2 Table). The comparisons between the different health insurance models were based on planned non-orthogonal contrasts: contrast 1: FPM vs. SCM, contrast 2: FPM-light vs. SCM, contrast 3: TM vs. SCM, contrast 4: FPM vs. FPM-light, contrast 5: FPM vs. TM. Corresponding confidence intervals (CI) and p-values were calculated based on simultaneous inference procedures to account for multiplicity. The procedure of multiple testing was performed using the multcomp package [39]. For all analyses the R programming language, version 4.1.0 (R Foundation for Statistical Computing) [40], was used.

### Ethics approval and informed consent

The study was based on retrospective, pre-existing, anonymized, and de-identified routine administrative healthcare claims data. Prior to the analysis, the data was anonymized and de-identified. According to the Swiss Federal Act on Research involving Human Beings (Human Research Act, HRA) [41] and the local ethics committee of the canton Zurich, ethical approval and written informed consent of patients was not required for the study. The data were accessed on 10.11.2022 for the research purposes.

## Results

Table 1 presents patient characteristics of the study samples regarding the different drug safety outcomes in 2021.

**Table 1:** Patient characteristics of the three populations of interest.

Sample	PIPPI	PIO	PIM/ Poly
	N (%)/ Mean (SD)	N (%)/ Mean (SD)	N (%)/ Mean (SD)
N	173’277	88’993	203’387
**Demographics**			
Sex			
Male	75’339 (43.5%)	32’720 (36.8%)	88’000 (43.3%)
Female	97’938 (56.5%)	56’273 (63.2%)	115’387 (56.7%)
Mean age	59.0 / (18.0)	59.1 / (18.6)	75.9 / (7.4)
Age groups (in years)			
18–24	6’089 (3.5%)	3’661 (4.1%)	
25–34	13’426 (7.7%)	6’615 (7.4%)	
35–44	19’994 (11.5%)	10’545 (11.8%)	
45–54	27’242 (15.7%)	14’558 (16.4%)	
55–64	33’649 (19.4%)	16’804 (18.9%)	
65–74	33’157 (19.1%)	14’881 (16.7%)	95’541 (47.0%)
75–84	28’797 (16.6%)	14’579 (16.4%)	78’197 (38.4%)
≥85	10’923 (6.3%)	7’350 (8.3%)	29’649 (14.6%)
**Language region**			
German-speaking area	132’269 (76.3%)	60’742 (68.3%)	155’275 (76.3%)
French-speaking area	27’897 (16.1%)	22’451 (25.2%)	31’480 (15.5%)
Italian-speaking area	12’805 (7.4%)	5’676 (6.4%)	16’236 (8.0%)
Rhaeto-Romanic-speaking area	306 (0.2%)	124 (0.1%)	396 (0.2%)
**Living area**			
City	117’264 (67.7%)	61’250 (68.8%)	132’798 (65.3%)
Rural	21’632 (12.5%)	10’785 (12.1%)	27’664 (13.6%)
Intermediate	34’381 (19.8%)	16’958 (19.1%)	42’925 (21.1%)
**Health insurance model**			
SCM	60’687 (35.0%)	32’483 (36.5%)	88’537 (43.5%)
FDM	71’773 (41.4%)	35’832 (40.3%)	80’077 (39.4%)
FDM-light	22’372 (12.9%)	12’591 (14.1%)	25’582 (12.6%)
TM	18’445 (10.6%)	8’087 (9.1%)	9’191 (4.5%)
**Number of chronic conditions**			
0–1	53’911 (31.1%)	15’555 (17.5%)	53’469 (26.3%)
2–3	55’549 (32.1%)	29’536 (33.2%)	82’704 (40.7%)
4–5	40’610 (23.4%)	26’204 (29.4%)	46’355 (22.8%)
≥6	23’207 (13.4%)	17’698 (19.9%)	20’859 (10.3%)
**Outpatient and Inpatient care**			
N outpatient consultations	13.4 / (12.1)	15.4 / (12.9)	11.6 / (10.0)
Hospitalization (≥1 overnight stay)	41’766 (24.1%)	26’077 (29.3%)	40’085 (19.7%)

Abbreviation: N, number; PIPPI, potentially inappropriate proton pump inhibitor; PIO, potentially inappropriate opioid; PIM, potentially inappropriate medication; SCM, standard care model; FDM, family doctor model; TM, telemedicine model.

Note: Outpatient consultations includes primary care and specialist physician.

Around 173’000 patients were eligible for the PIPPI sample. More than half of the sample were women (57%), and the average age was 59 years. Almost 80% of the sample lived in the German-speaking part of Switzerland and almost 70% in urban areas. 65% were enrolled in an ICM. Almost 70% suffered from two or more chronic conditions. Patients had on average 13 consultations with GPs and specialists and about one-fourth were hospitalized in 2021.

About 89’000 patients were included in the PIO sample, where almost two thirds were women (63%) and with an average age of 59 years. Again, most patients lived in the German-speaking part of Switzerland (68%) and in urban areas (69%), chose more frequent an ICM than a SCM (ICM: 63.5%; SCM: 36.5%), and were multimorbid (83%). Patients had on average 13 consultations and almost one third of this patient group was hospitalized (30%).

The PIM and polypharmacy sample consists of a total of 203’387 elderly patients. Almost 60% were women, the average age was 76 years. Three quarter lived in the German-speaking part of Switzerland (76%), nearly two-thirds (65%) lived in urban areas, and more than half choose an ICM. Approximately 75% had two or more chronic conditions. Elderly patients had on average 12 consultations and 20% of them were hospitalized.

Table 2 presents the one-year prevalence of drug safety outcomes by different health insurance models. Approximately 30% and 20% of the patients in SCM received PIPPI and PIO, respectively. Prevalence was substantially lower in FDM (PIPPI: 22%; PIO: 14%), FDM-light (PIPPI: 24%; PIO: 15%) and TM (PIPPI, PIO: 10%). The lowest prevalence of PIM prescription was shown in elderly patients enrolled in TM (26%) followed by FDM (30%), SCM (34%) and FDM-light (37%). Polypharmacy was also observed less prevalent in elderly patients enrolled in TM (34%) and FDM (48%) than in SCM and FDM-light (both 53%).

**Table 2:** Prevalence of drug safety outcomes by health insurance models.

Drug safety		Health insurance model			
	Total	SCM	FDM	FDM-light	TM
	N (%)	N (%)	N (%)	N (%)	N (%)
PIPPI	42’180 (24.3)	18’844 (31.1)	15’994 (22.3)	5’445 (24.3)	1’897 (10.3)
PIO	14’706 (16.5)	6’807 (21.0)	5’170 (14.4)	1’933 (15.4)	796 (9.8)
PIM	66’608 (32.7)	30’469 (34.4)	24’237 (30.3)	9’481 (37.1)	2’421 (26.3)
Polypharmacy	102’196 (50.2)	47’016 (53.1)	38’433 (48.0)	13’592 (53.1)	3’155 (34.3)

Abbreviation: N, number; PIPPI, potentially inappropriate proton pump inhibitor; PIO, potentially inappropriate opioid; PIM, potentially inappropriate medication; SCM, standard care model; FDM, family doctor model; TM, telemedicine model.

**Fig 1:**
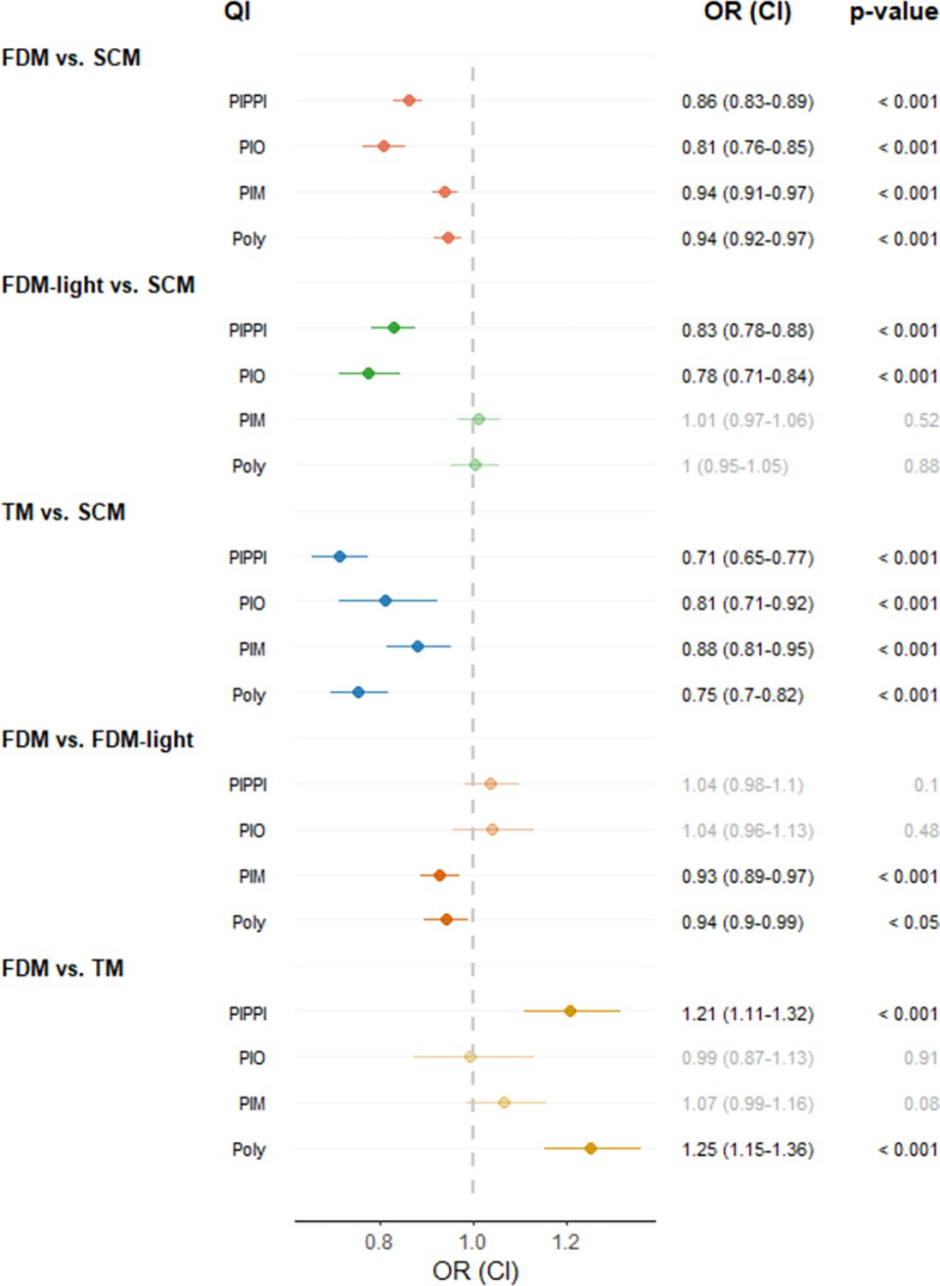
Estimated association between health insurance models and the drug safety outcomes. Abbreviation: OR, odds ratio; CI, confidence interval; PIPPI, potentially inappropriate proton pump inhibitor; OPI, potentially inappropriate opioid; PIM, potentially inappropriate medication; Poly, polypharmacy; SCM, standard care model; FDM, family doctor model; TM, telemedicine model. Notes: The estimated associations were based on propensity score weighted regression analyses, additionally controlled for covariates: Age; sex; language region; number of pharmaceutical cost groups, outpatient consultations and hospitalization (assessed one year prior of the observation year of the drug safety outcomes (2020)), showing all significant estimates (results not shown). CI and p-values were calculated based on simultaneous inference procedures to take multiplicity into account.

Fig 1 displays the estimated association between health insurance models and drug safety outcomes from the weighted multiple logistic regression models. Patients who were enrolled in FDM had statistically significant lower odds of receiving PIPPI (OR, 0.86; CI 95%, 0.83–0.89), PIO (OR, 0.81; CI 95%, 0.76–0.85), PIM (OR, 0.94; CI 95%, 0.91–0.97), and polypharmacy (OR, 0.94; CI 95%, 0.92–0.97) than patients in SCM. The odds of receiving potentially inappropriate prescribing were also significantly lower for patients who were enrolled in TM and partly in FDM-light compared to patients enrolled in SCM. Moreover, elderly patients enrolled in FDM showed lower odds of receiving PIM (OR, 0.93; CI 95%, 0.89–0.97) and polypharmacy (OR, 0.94; CI 95%, 0.90–0.99) than elderly in FDM-light. There was no statistically significant difference in receiving PIPPI and PIO between patients in FDM and FDM-light. Furthermore, patients enrolled in FDM had higher odds of receiving PIPPI (OR, 1.21; CI 95, 1.11–1.32) than patients in TM. The odds were also higher for elderly patients in FDM to receive polypharmacy (OR, 1.25; CI 95%, 1.15–1.36) compared to those in TM. There was no difference in prescription of PIO and PIMs between patients in FDM and TM.

## Discussion

To the best of our knowledge, this is the first study in Switzerland providing comprehensive information on the comparison between patients in different health insurance models regarding their drug safety.

Our study revealed three main findings. First, the probability of receiving potentially inappropriate prescribing was lower in patients enrolled in ICMs compared to SCM for most outcomes. This finding is in line with previous Swiss studies showing lower rates of inappropriate prescribing in different ICMs than SCM [23,24]. Similar results were also shown in a German study which evaluated the implementation of the German ICM in the state of Baden-Württemberg. Medications with high-quality and cost-effective alternatives were significantly less prescribed among patients enrolled in the ICM compared to patients not enrolled in the model [42].

Elements of IC such as gatekeeping and coordination of care may result in a close and continuing relationship between physician and their patients including a detailed knowledge on patients’ medical history and prescribed medication from other healthcare providers. Thereby, IC have likely strengthened the awareness of mis-, under- and over-prescription of medication of the treating physician. Surprisingly, in elderly patients enrolled in FDM-light, we found no evidence for lower levels of exposure to PIM and polypharmacy compared to those in SCM. One explanation could be the poorer degree of gatekeeping and coordination of care in FDM-light. Compared to other ICMs, the provision of IC is not financially covered and there is no restriction for the prescription of certain medications. Especially for PIM in elderly patients, where the medication plan is often highly complex, special attention to coordinated care is required.

Second, elderly patients enrolled in FDM revealed lower odds for receiving PIM prescription and polypharmacy than elderly patients in FDM-light. This might have various reasons. In contrast to FDM-light, FDM is the most comprehensive ICM, where physicians part of a physician network following network-specific quality standards and with a financial-based cooperation agreement strengthening the provision of IC services.

In addition, in 2020 an incentive scheme was implemented in the established agreement between Helsana Group and physician networks to improve drug safety regarding the prescription of PIM and polypharmacy in patients enrolled in FDM. The effectiveness of this incentive scheme on diabetes guidelines adherence has been shown in a recently published evaluation [43]. Therefore, it can be assumed that the introduction of the incentive scheme had additionally influenced the prescription habits of PIM and polypharmacy in patients enrolled in FDM.

Furthermore, previous studies revealed beneficial effects of chronic care management (CCM) programs on the treatment of various chronic diseases [44–46]. Since CCM programs and FDM both incorporate key aspects of IC and the chronically ill patients are highly prevalent in older populations, we assume that a huge part of them might have particularly benefitted from FDM, also regarding drug safety. In this context, Laux et al. also highlighted the beneficial influence of ICM for chronically ill and elderly patients and showed a significant reduction in two indicators relevant for older and chronically ill patients: the prescription of antipsychotics (particularly for dementia) and the long-term prescription of benzodiazepines [42].

Third, patients enrolled in FDM had higher odds of receiving PIPPI prescription and polypharmacy than patients in TM. One explanation could be the stronger patient-centered function of medical centers for medication prescription. Medications are only prescribed for defined medical indications and repeat prescriptions where indication and dosage are clearly declared and documented. Therefore, a more restrictive prescribing behavior might have resulted in less inadequate prescribing.

Furthermore, there were no differences in the prescribing of PIO and PIM between FDM and TM. This finding might suggest a high degree of awareness for drug safety in opioids and PIM prescriptions among patients enrolled in both FDM and TM.

Overall, the present study provides updated and extended information on the association between ICMs with different level of IC and drug safety outcome in terms of inappropriate prescribing of frequently used medications, like PPI, or even highly addictive substances, such as opioids.

Our findings reveal that ICMs with a high degree of coordination and gatekeeping may generate substantial patient benefits, including a reduction in inappropriate prescribing and the prevention of associated risks of drug-related adverse events, like hospitalizations or even deaths. Thus, ICMs can potentially reduce avoidable healthcare costs and loss of quality of life. In the context of coordinated care, interprofessional collaboration involving clinical pharmacologist and pharmacist may further help to reduce and prevent inappropriate prescribing [47]. This is especially valuable during transition between healthcare sectors, such as from hospital to outpatient care, when changes of medication regime may likely occur, and accurate medication reconciliation is essential.

Additionally, in periods of skilled labor shortages and high clinician workloads, the integration of data-based automatic detection systems to identify potential inadequate prescribing is highly desirable [48,49]. Although not on an individual patient level, but on the level of physician networks, such systems have been applied to assess the prevalence of PIM and polypharmacy for benchmarking as part of the incentive scheme within the cooperation agreement. However, this concept applies only to the FDM and not to any of the other insurance models, and due to strict data protection regulations, an application on the individual patient level is currently not feasible in Switzerland.

More research is needed to focus on specific aspects of IC in different populations. This is particularly important in the context of an aging population and the rising prevalence of chronic diseases, in which extensive medication use is highly prevalent.

### Strengths and limitations

The study has several strengths and limitations. One of the main strengths are the large sample sizes based on health insurance claims data representing practice-based information including comprehensive information on medication and received healthcare services. In addition, we applied a doubly robust method for the evaluation of the association between different health insurance models and drug safety outcomes. The method combines regression adjustments with weights based on propensity scores to obtain more robust and consistent estimators.

The study has also some notable limitations. First, patients in FDM or FDM-light may be treated by the same physician as patients in SCM or TM, because physicians are not exclusively assigned to one health insurance model. This limitation might bias the association between ICMs and outcomes of drug safety. However, previous studies investigating the efficiency of FDM and FDM-light revealed differences in provision of care depending on the patients’ health insurance models. For example, patients enrolled in SCM reported lower efficiency of care than patients enrolled in FDM, although the treating physician was part of a physician network in both cases [50].

Second, we cannot exclude selection bias of high performing physicians in physician networks. It is on a voluntary basis to join a physician network and to fulfill the required network specific quality standards. Maybe physicians who were already providing high quality care tend to fulfill the quality standards and join the physician network.

Third, there might also be a selection bias in the choice of the health insurance model. Although we carefully adjusted our estimated models for potential patient-level confounders within the given samples, we cannot exclude biases through unobserved confounders which might have influenced the choice of the health insurance model.

Fourth, database and study design do not allow for causal inferences about the effect of ICMs on the quality of care (as measured by drug safety outcomes). However, sensitivity analyses showed that applied doubly robust procedures improved the overall model performance, and thus, suggests reduced bias from potential confounders (S2 Table).

Finally, our findings may not be representative of the entire Swiss population. However, this study was based on a large nationwide health insurance claims data covering more than one fifth of the total population with mandatory health insurance across all geographical regions of Switzerland.

## Conclusion

ICMs were significantly associated with higher drug safety compared to SCM for most outcomes. Findings suggest that patients may benefit most from ICMs with a high degree of coordination or gatekeeping. ICM may represent an effective approach to improve patients’ drug safety and, thus, to reduce the risk of adverse events.

## Supporting information

S1 Table Description of the drug safety outcomes. (DOCX)

S2 Table Estimated association between health insurance models and drug safety outcomes using three different modelling approaches. (DOCX)
