# Supplementary material for: Are integrated care models associated with improved drug safety in Swiss primary care? an observational analysis using healthcare claims data
Source: PLoS One. 2024 Sep 26;19(9):e0311099. doi: 10.1371/journal.pone.0311099 (PMC11426503; doi:10.1371/journal.pone.0311099)
Supplement: S1 Table — (DOCX) [file pone.0311099.s001.docx]

**S1 Table. Description of the drug safety outcomes**

| **Outcome** | **Measure** | **Study population** | **Comments** |
| --- | --- | --- | --- |
| Potentially inappropriate use of proton pump inhibitor (PIPPI) | Is an individual exposed to PIPPI in the given year: yes/no | Insured persons with the prescription of ≥1 proton pump inhibitor(s) (PPI) in the given year | Operationalization is based on Muheim et al. 2021 |
| Potentially inappropriate use of opioids (PIO) | Is an individual exposed to PIO in the given year: yes/no | Insured persons with ≥1 prescription(s) of pain medications | The identification of pain medication and weak opioids were based on ATC classification. Pain medication: nonsteroidal anti-inflammatory drugs (M01AA, M01AB, M01AC, M01AE, M01AG), cox-inhibitors (M01AH), metamizole (N02BB02, N02BB52, N02BB72), paracetamol (N02BE01, N02BE51, N02BE71). Weak opioids: codeine and combinations (N02AA59), tilidine (N02AX01), tramadol (N02AX02) and tapentadol (N02AX06) |
| Potentially inappropriate medication (PIM) | Is an individual exposed to ≥1 PIM per quarter year aggregated at the annual level | Insured persons aged 65 years or older with ≥1 prescribed medication in the given year | Based on ATC codes and PRISCUS and Beers criteria |
| Polypharmacy | Is an individual prescribed ≥5 different active ingredients (i.e., ATC codes) per quarter year aggregated at the annual level |  |  |
