# Supplementary material for: Are integrated care models associated with improved drug safety in Swiss primary care? an observational analysis using healthcare claims data
Source: PLoS One. 2024 Sep 26;19(9):e0311099. doi: 10.1371/journal.pone.0311099 (PMC11426503; doi:10.1371/journal.pone.0311099)
Supplement: S2 Table — (DOCX) [file pone.0311099.s002.docx]

**S2 Table. Estimated association between health insurance models and drug safety outcomes using three different modelling approaches**

|  | **Option I: simple GLM** | | **Option II: multiple GLM** | | **Option III: PSW GLM** | |
| --- | --- | --- | --- | --- | --- | --- |
|  | **OR (CI)** | **P-Value** | **OR (CI)** | **P-Value** | **OR (CI)** | **P-Value** |
| **FDM vs. SCM** |  |  |  |  |  |  |
| PIPPI | 0.64 (0.62 – 0.66) | 0.0000 | 0.87 (0.84 – 0.90 | 0.0000 | 0.86 (0.83 – 0.89) | 0.0000 |
| PIO | 0.64 (0.6 – 0.67) | 0.0000 | 0.82 (0.78 – 0.87) | 0.0000 | 0.81 (0.76 – 0.85) | 0.0000 |
| PIM | 0.83 (0.81 – 0.85) | 0.0000 | 0.94 (0.92 – 0.97) | 0.0000 | 0.94 (0.91 – 0.97) | 0.0000 |
| Polypharmacy | 0.82 (0.80 – 0.84) | 0.0000 | 0.94 (0.92 – 0.97) | 0.0000 | 0.94 (0.92 – 0.97) | 0.0000 |
| **FDM-light vs. SCM** |  |  |  |  |  |  |
| PIPPI | 0.71 (0.68 – 0.75) | 0.0000 | 0.87 (0.82 – 0.91) | 0.0000 | 0.83 (0.78 – 0.88) | 0.0000 |
| PIO | 0.68 (0.64 – 0.73) | 0.0000 | 0.82 (0.76 – 0.88) | 0.0000 | 0.78 (0.71 – 0.84) | 0.0000 |
| PIM | 1.12 (1.08 – 1.16) | 0.0000 | 1.01 (0.97 – 1.05) | 0.4181 | 1.01 (0.97 – 1.06) | 0.5184 |
| Poly | 1.00 (0.97 – 1.04) | 0.9373 | 0.98 (0.94 – 1.02) | 0.2195 | 1.00 (0.95 – 1.05) | 0.8821 |
| **TM vs. SCM** |  |  |  |  |  |  |
| PIPPI | 0.25 (0.24 – 0.27) | 0.0000 | 0.69 (0.64 – 0.74) | 0.0000 | 0.71 (0.65 – 0.77) | 0.0000 |
| PIO | 0.41 (0.37 – 0.45) | 0.0000 | 0.82 (0.74 – 0.91) | 0.0000 | 0.81 (0.71 – 0.92) | 0.0002 |
| PIM | 0.68 (0.64 – 0.72) | 0.0000 | 0.89 (0.84 – 0.95) | 0.0000 | 0.88 (0.81 – 0.95) | 0.0002 |
| Polypharmacy | 0.46 (0.44 – 0.49) | 0.0000 | 0.75 (0.70 – 0.80) | 0.0000 | 0.75 (0.70 – 0.82) | 0.0000 |
| **FDM vs. FDM-light** |  |  |  |  |  |  |
| PIPPI | 0.89 (0.85 – 0.93) | 0.0000 | 1.00 (0.95 – 1.06) | 0.9010 | 1.04 (0.98 – 1.1) | 0.1035 |
| PIO | 0.93 (0.86 – 1.00) | 0.0117 | 1.01 (0.93 – 1.09) | 1.0000 | 1.04 (0.96 – 1.13) | 0.4847 |
| PIM | 0.74 (0.71 – 0.77) | 0.0000 | 0.93 (0.89 – 0.97) | 0.0000 | 0.93 (0.89 – 0.97) | 0.0002 |
| Polypharmacy | 0.81 (0.79 – 0.84) | 0.0000 | 0.96 (0.92 – 1.01) | 0.0936 | 0.94 (0.90 – 0.99) | 0.0058 |
| **FDM vs. TM** |  |  |  |  |  |  |
| PIPPI | 2.50 (2.34 – 2.67) | 0.0000 | 1.27 (1.18 – 1.36) | 0.0000 | 1.21 (1.11 – 1.32) | 0.0000 |
| PIO | 1.54 (1.40 – 1.71) | 0.0000 | 1.00 (0.90 – 1.12) | 1.0000 | 0.99 (0.87 – 1.13) | 0.9137 |
| PIM | 1.21 (1.14 – 1.29) | 0.0000 | 1.05 (0.99 – 1.13) | 0.0837 | 1.07 (0.99 – 1.16) | 0.0826 |
| Polypharmacy | 1.77 (1.67 – 1.87) | 0.0000 | 1.26 (1.18 – 1.35) | 0.0000 | 1.25 (1.15 – 1.36) | 0.0000 |
| **AUC Values** |  |  |  |  |  |  |
|  | **Option I: simple GLM** | | **Option II: multiple GLM** | | **Option III: PSW GLM** | |
| PIPPI | 0.59 | | 0.80 | | 0.80 | |
| PIO | 0.57 | | 0.71 | | 0.71 | |
| PIM | 0.53 | | 0.69 | | 0.69 | |
| Polypharmacy | 0.54 | | 0.83 | | 0.83 | |
| Abbreviation: GLM, generalized linear model; PSW, propensity score weighting; OR, odds ratio; CI, confidence interval; AUC, area under the curve; PIPPI, potentially inappropriate proton pump inhibitors; PIO, potentially inappropriate opioids; PIM, potentially inappropriate medications; Poly, polypharmacy; SCM, standard care model; FDM, family doctor model; TM, telemedicine model.  Notes: Estimated association between health insurance models and drug safety outcomes, using three different modelling approaches. For each outcome and modelling approach 1 regression model was calculated, subsequently sorted by the estimates of health insurance models represents a single statistical model. Option I and II represent sensitivity analysis. Sample sizes: PIPPI: 173’277; PIO: 88’993; PIM and polypharmacy: 203’387. Option II & III: models are additionally controlled for age, sex, language region number of PCGs and number of outpatient consultations, all showing significant estimates (results not shown). | | | | | | |
